# A Rare Case of Comorbidity Between Intravascular Large B-Cell Lymphoma and Small-Cell Lung Cancer

**DOI:** 10.7759/cureus.68969

**Published:** 2024-09-09

**Authors:** Sayato Fukui, Yoshimasa Kura

**Affiliations:** 1 Department of General Medicine, Juntendo University Faculty of Medicine, Tokyo, JPN; 2 Department of Hematology, Kasukabe Municipal Medical Center, Kasukabe, JPN

**Keywords:** biopsy, lung cancer, malignant lymphoma, multiple cancers, soluble interleukin-2 receptor

## Abstract

We report the case of a 60-year-old male with a fever for two months and a skin rash for approximately one month prior to visiting his local doctor and subsequent admission to the hospital. Clinical findings included fever, weight loss, and night sweats. Computed tomography (CT) revealed an irregularly shaped mass bordering the upper lobe of the left lung and mediastinum, as well as hepatosplenomegaly. Suspecting lung cancer or malignant lymphoma, the patient was referred to our hospital for further evaluation. Positron emission tomography-computed tomography (PET/CT) revealed hepatosplenomegaly with accumulation of contrast agents in the liver, spleen, and bone marrow, as well as in a mass in the left upper lobe. A liver biopsy revealed atypical cells in the sinusoids, and immunohistochemical staining confirmed B-cell lymphoma. Chemotherapy was initiated immediately. PET/CT at the follow-up evaluation showed that the hepatosplenomegaly and bone marrow-related accumulation of contrast agents had resolved, but the accumulation of contrast agents in the mediastinal lymph nodes and the left upper lobe mass persisted, despite shrinkage. A bronchoscopy and mediastinal lymph node biopsy were performed. Histopathological examination revealed that the lung mass was most likely a small-cell carcinoma of the lung. Clinically, the malignant lymphoma was considered intravascular large B-cell lymphoma. As a result of appropriate treatment for both cancers, the patient’s survival period improved.

## Introduction

Malignant lymphoma and lung cancer are treated with hematology and respiratory medications, respectively. According to a report from the National Cancer Institute in Japan, in 2019, malignant lymphoma had an incidence of 29.0 cases per 100,000 people, while lung cancer had an incidence of 100.3 cases per 100,000 people [1]. Reports of cancers overlapping with other malignancies (solid tumors) and malignant lymphomas do exist (e.g., overlapping breast cancer and malignant lymphoma) [2]. Although there is some literature showing the coexistence of lung cancer and malignant lymphoma [3-7], there are no reports of cases involving intravascular large B-cell lymphoma (IVLBCL) and small-cell carcinoma of the lung, especially in this case. Similarly, although there are very few reports of overlapping cancers, particularly malignant lymphomas and solid tumors, there are some reports of patients developing solid tumors after treatment for malignant lymphoma [8,9].

This report, which was previously presented as a meeting abstract at the 80th Annual Meeting of the Japanese Society of Hematology on October 12, 2018, describes a rare case of malignant lymphoma overlapping with lung cancer.

## Case presentation

A 60-year-old male presented with a fever that had persisted for two months. The fever spiked during the night, with a maximum temperature of about 39°C. Acetaminophen was taken as needed for the fever. Additionally, he had a skin rash for approximately one month prior to visiting his local doctor. He was admitted to the hospital after a thorough examination, as he was also found to have hepatosplenomegaly. Clinical findings included fever, weight loss of about 8 kg over two months, and night sweats. Respiratory symptoms were limited to a mild cough.

Computed tomography (CT) revealed an irregularly shaped mass bordering the upper lobe of the left lung and mediastinum, as well as hepatosplenomegaly. Suspecting lung cancer or malignant lymphoma, the patient was referred to our hospital for admission.

**Figure 1:**
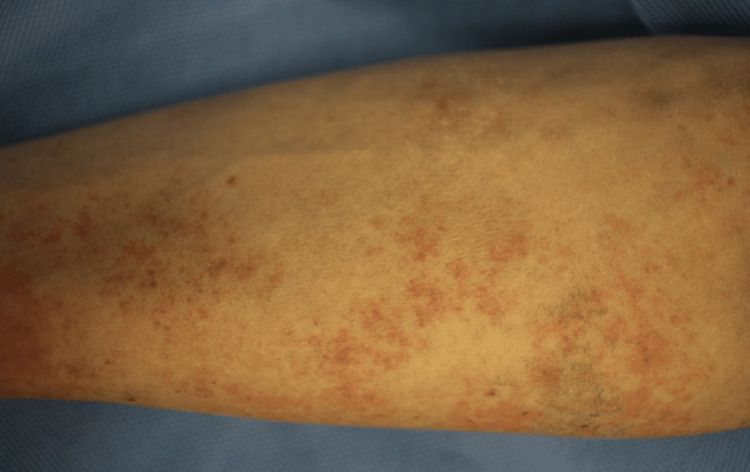
Photograph showing fine erythema on the trunk (disseminated erythema rubrum and petechial purpura on the trunk and extremities)

The patient had no specific pre-existing medical conditions, and his chief complaints were fever, weight loss, and night sweats. His vital signs on admission were as follows: clear consciousness; a temperature of 37.8°C; blood pressure of 124/81 mmHg; pulse of 85 beats per minute; respiratory rate of 18 breaths per minute; and SpO2 of 98%. Physical examination revealed hepatosplenomegaly and fine erythema, mainly on the trunk (disseminated erythema rubrum and petechial purpura on the trunk and extremities), as shown in Figure 1.

Blood biochemistry tests revealed anemia (hemoglobin = 11.5 g/dL), thrombocytopenia (platelet count = 46,000/µL), elevated lactate dehydrogenase (610 IU/L), and elevated C-reactive protein (22.32 mg/dL), as shown in Table 1.

**Table 1:** Results of blood tests on admission IU: International unit; Eq: Equivalent; s: Second

Blood test	Result	Normal range
White blood cells (/μL)	4,030	4,000-8,000
Neutrophils (%)	36.0	40.0-75.0
Lymphocytes (%)	48.0	30.0-50.0
Atypical-lymphocytes (%)	5.0	0.0
Hemoglobin (g/dL)	11.5	12.0-16.0
Platelets (/μL)	4.6 × 10^4^	13.0 × 10^4^-35.0 × 10^4^
Total bilirubin (mg/dL)	1.4	0.2-1.0
Aspartate aminotransferase (IU/L)	38	12.0-32.0
Alanine aminotransferase (IU/L)	15	8.0-36.0
Lactate dehydrogenase (IU/L)	610	127.0-221.0
Blood urea nitrogen (mg/dL)	33.7	8.0-20.0
Creatinine (mg/dL)	1.14	0.3-1.2
Sodium (mEq/L)	137	134.0-147.0
Potassium (mEq/L)	4.5	3.2-4.8
Chloride (mEq/L)	100	98-108
C-reactive protein (mg/dL)	22.32	0.0-0.3
Soluble interleukin-2 receptor (U/mL)	14,500	0-587
Neuron-specific enolase (ng/mL)	28.9	0-16.3
Pro-gastrin-releasing peptide (pg/mL)	107	0-81.0
Prothrombin time (s)	14.0	9.5-13.5
Activated partial thromboplastin time (s)	49.4	26.0-38.0

**Figure 2:**
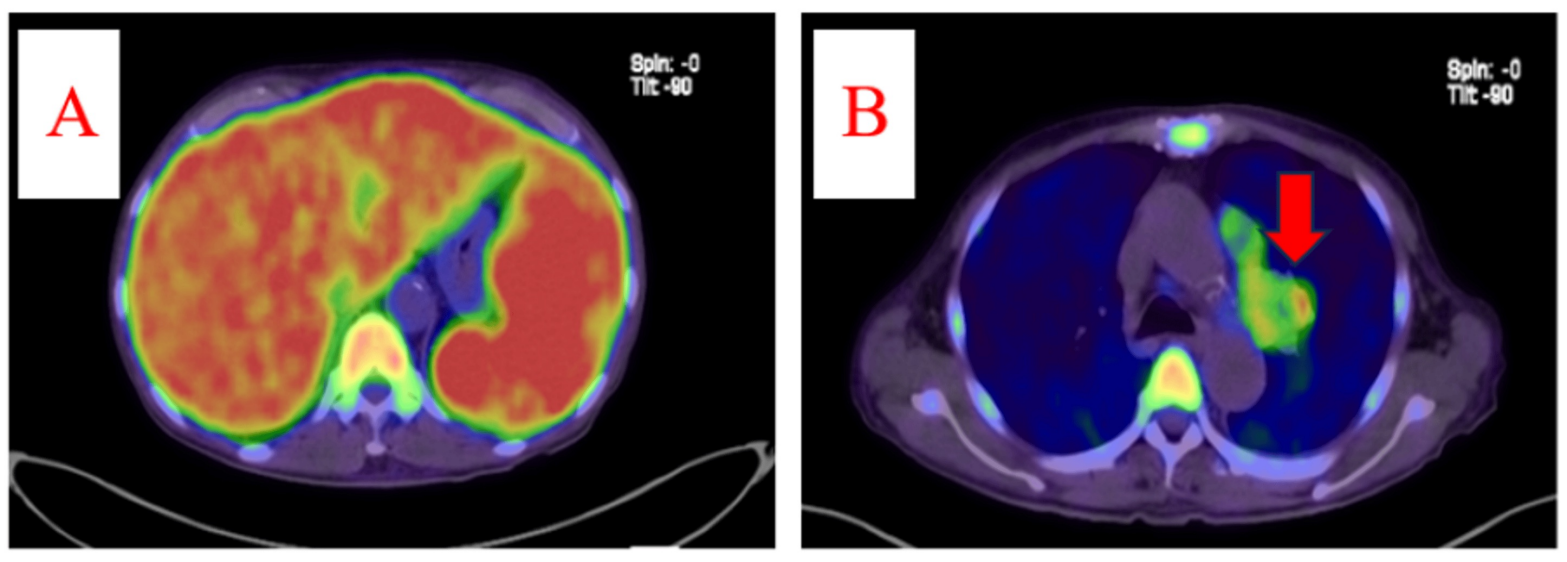
Positron emission tomography/computed tomography (PET/CT) showing (A) hepatosplenomegaly, with accumulation of contrast agent in the liver, spleen, and bone marrow, and (B) accumulation of contrast agent in a mass in the left upper lobe (red arrow)

Visual examination of the hemogram revealed approximately 5% atypical lymphocytes in the peripheral blood. Furthermore, tumor markers showed elevated levels of soluble interleukin-2 receptor (sIL-2R = 14,500 U/mL), neuron-specific enolase (28.9 ng/mL), and pro-gastrin-releasing peptide (107 pg/mL). Positron emission tomography-computed tomography (PET/CT) revealed hepatosplenomegaly with accumulation of contrast agents in the liver, spleen, and bone marrow, as well as in a mass in the left upper lobe (Figure 2).

**Figure 3:**
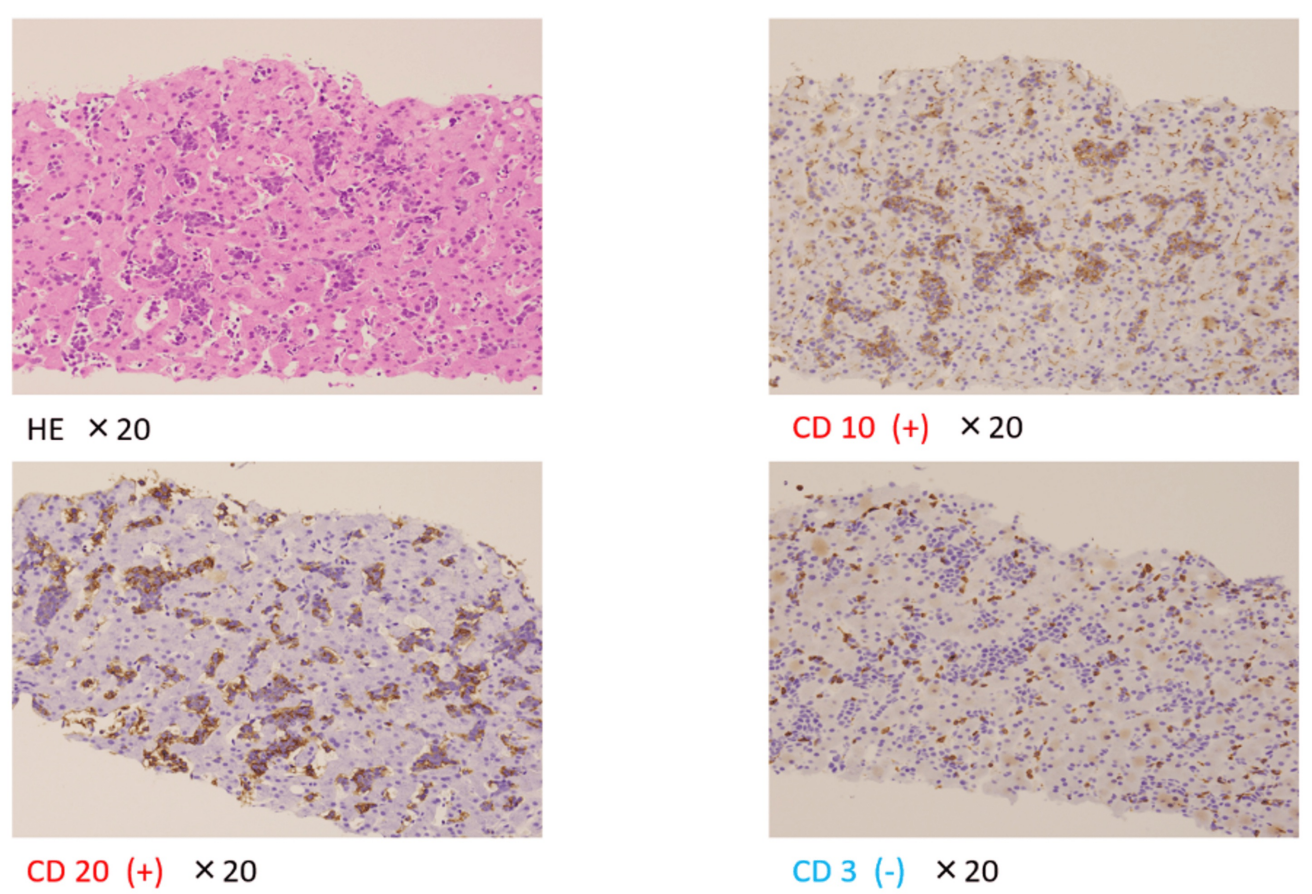
Immunohistochemical staining was negative for cluster of differentiation (CD) 3, negative for CD 5, positive for CD 10, and positive for CD 20, leading to a confirmed diagnosis of B-cell lymphoma HE: Hematoxylin-eosin stain

Bone marrow aspiration was performed as soon as possible due to the rapid progression of the disease. Despite a result of 8.8% abnormal lymphocytes, a definitive diagnosis was not reached. A skin biopsy at the site of the rash was performed; however, no significant findings were obtained. Subsequently, a liver biopsy was performed, which revealed atypical cells in the sinusoids. Immunohistochemical staining was negative for cluster of differentiation (CD) 3, negative for CD 5, positive for CD 10, and positive for CD 20, leading to a confirmed diagnosis of B-cell lymphoma (Figure 3).

Six courses of rituximab, cyclophosphamide, doxorubicin, vincristine, and prednisolone (R-CHOP) were administered, and high-dose methotrexate was given for central nervous system prophylaxis. PET/CT at the follow-up evaluation showed that hepatosplenomegaly- and bone marrow-related accumulation of contrast agent had resolved, but the accumulation of contrast agent persisted in the mediastinal lymph nodes and left upper lobe mass.

**Figure 4:**
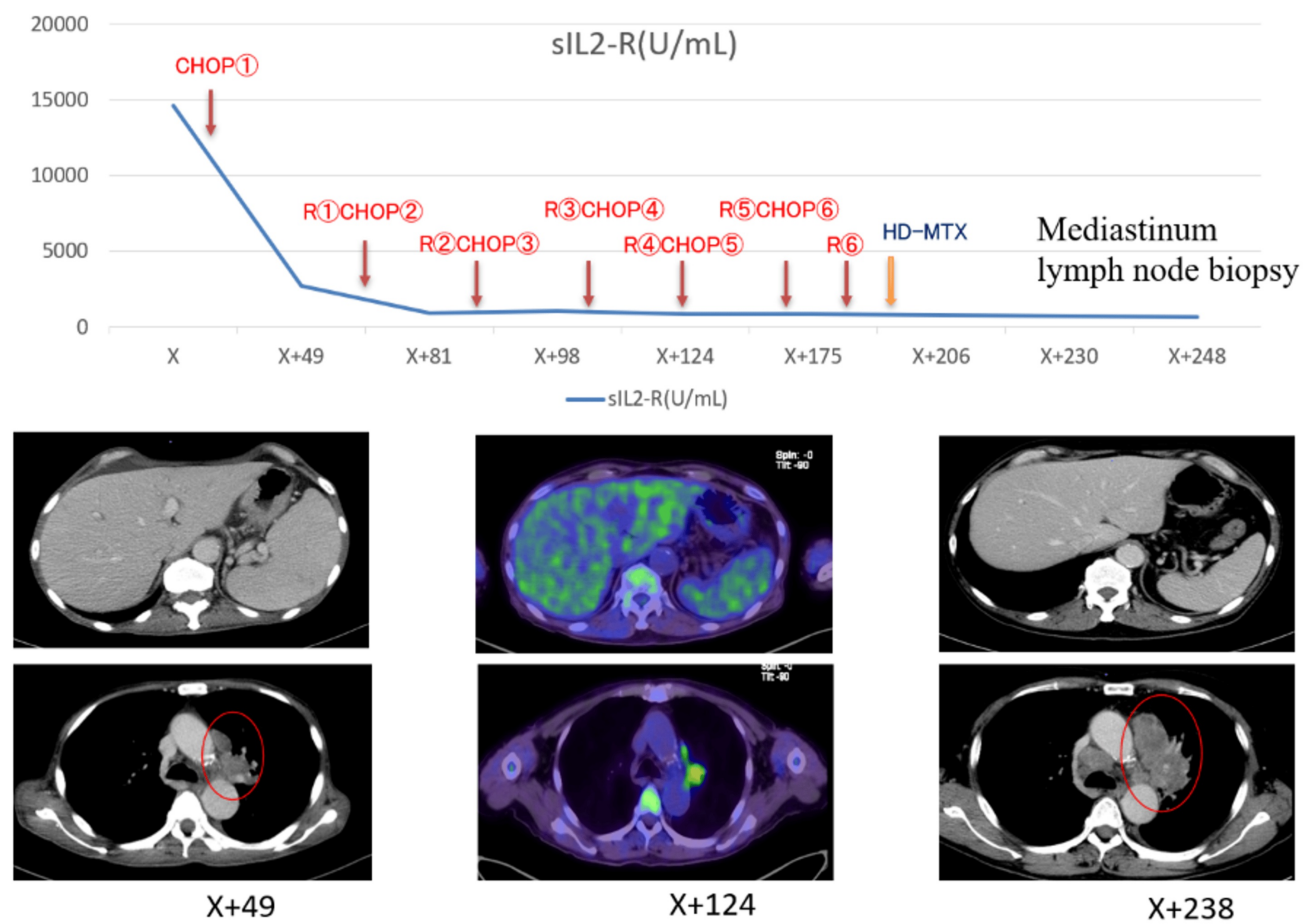
Overall course of treatment and changes in positron emission tomography/computed tomography (PET/CT) and soluble interleukin-2 receptor (sIL2-R) Post-treatment CT revealed that the mediastinal lymph nodes and left upper lobe mass continued to increase in size (red circle). sIL2-R: Soluble interleukin-2 receptor; RCHOP: Rituximab-cyclophosphamide, doxorubicin, vincristine, prednisolone; HD-MTX: High-dose methotrexate

Post-treatment CT revealed that the mediastinal lymph nodes and left upper lobe mass continued to increase in size. The sIL-2R level, which was high at initial onset, did not increase after treatment, and hepatosplenomegaly was not present; therefore, the mediastinal lymph nodes and left upper lobe mass were suspected to be associated with another disease (Figure 4).

A bronchoscopy and mediastinal lymph node biopsy were performed, with histopathological examination revealing that the lung lesion was most likely a Stage III small-cell carcinoma of the lung (neuron-specific enolase and pro-gastrin-releasing peptide were positive, as shown in Table 1). Clinically, the malignant lymphoma was considered an IVLBCL (International Prognostic Index was Stage IVB).

## Discussion

This report describes a rare case of overlapping malignant lymphoma (IVLBCL) and lung cancer (small-cell carcinoma). Reports of cancers overlapping with other malignancies (solid tumors) and malignant lymphomas do exist, such as overlapping breast cancer and malignant lymphoma [2]. Although there is some literature showing the coexistence of lung cancer and malignant lymphoma [3-7], there are no reports of cases involving IVLBCL and small-cell carcinoma of the lung, especially in this case report.

However, there have been many reports of lung cancer developing after treatment for malignant lymphoma as a secondary cancer. This phenomenon is thought to be related to the effects of chemotherapy on malignant lymphomas. One meta-analysis reported a significantly higher incidence of lung cancer after treatment for Hodgkin lymphoma [8,9]. Studies have also reported an association between survivors of malignant lymphoma and the development of breast cancer; however, a causal relationship remains unclear [10].

It should be noted that it is difficult to distinguish lung cancer from malignant lymphoma on imaging, even though they are not overlapping cancers. Reports exist of rare cases of B-cell lymphoma with tumor thrombi in the pulmonary veins and left atrium, and imaging findings suggestive of metastatic lung cancer [11]. In particular, it has been reported that among lung cancers, small-cell carcinoma is difficult to distinguish from lymphoma, even with PET-CT [12]. A biopsy is useful for differentiation; however, in the present case, it was clinically difficult to biopsy the mediastinal mass before treating the malignant lymphoma because of the rapid deterioration in the patient's general condition.

The treatment of patients with multiple cancers, as in this case, has not yet been established in the literature and should be considered based on the severity of each patient's disease [3-6]. Usually, the malignancy considered life-threatening should be treated first. The patient was first treated for malignant lymphoma, and his general condition improved; therefore, appropriate treatment was administered for the lung cancer diagnosed by biopsy, which further prolonged his life.

## Conclusions

In this case report, the patient was diagnosed with a rare case of overlapping malignant lymphoma and lung cancer. Malignant lymphoma was first diagnosed by biopsy, and appropriate treatment was subsequently provided, leading to the diagnosis and treatment of lung cancer.

This case is rare because of the coexistence of intravascular large B-cell lymphoma and small-cell lung cancer. The treatment of patients with multiple cancers, as in this case, has not yet been established in the literature and should be considered based on the severity of each patient's disease. In cases of coexisting malignancies, healthcare providers must carefully follow the course of each malignancy after its treatment.
